# Redlining, reinvestment, and racial segregation: a bayesian spatial analysis of mortgage lending trajectories and firearm-related violence

**DOI:** 10.1186/s40621-025-00579-9

**Published:** 2025-05-02

**Authors:** Gia Barboza-Salerno, Brittany Liebhard, Sharefa Duhaney, Taylor Harrington

**Affiliations:** 1 College of Social Work, The Ohio State University, Columbus, OH USA; 2 College of Public Health, The Ohio State University, 1841 Neil Avenue 352 Cunz Hall, Columbus, 43210 OH USA

**Keywords:** Housing discrimination, Bayesian spatial model, Gun violence, Area deprivation, HMDA

## Abstract

**Background:**

In the United States, firearm-related violence disproportionately impacts low-income, racially segregated communities more than affluent, predominantly White neighborhoods. This trend stems from historical disinvestment, discriminatory lending practices, and persistent structural inequalities. Housing policies have enforced racial segregation, limiting wealth accumulation in low-income communities. This study examines the relationship between historical and contemporary lending discrimination in mortgage originations and firearm-related violence in Chicago, Illinois. By analyzing investment and disinvestment patterns, we assess how housing discrimination continues to influence the risk of victimization in various social contexts.

**Methods:**

Redlining scores were derived from the 1930s Homeowners’ Loan Corporation (HOLC) grades, while contemporary lending indicators were obtained from the 2019 Home Mortgage Disclosure Act (HMDA). We classified neighborhoods into four lending trajectories—sustained disinvestment, disinvestment, growing investment, and high investment—based on historical redlining and contemporary mortgage lending patterns. Sustained disinvestment reflects historical redlining and ongoing lending discrimination, while growing investment targets areas that were historically redlined but are now experiencing increased lending activity. Bayesian spatial models examined firearm-related homicide risk across lending trajectories, adjusting for area deprivation index (ADI) and racial segregation.

**Results:**

In unadjusted models, sustained disinvestment (Relative Risk [RR] = 2.230, 95% CrI: [1.352, 3.681]) was associated with increased firearm-related homicide risk, while growing investment (RR = 0.782, 95% CrI: [0.452, 1.359]) and high investment (RR = 0.146, 95% CrI: [0.054, 0.397]) were associated with lower risk. After adjusting for ADI and racial segregation, the effect of sustained disinvestment attenuated (RR = 1.714, 95% CrI: [1.054, 2.791]), suggesting partial mediation. However, growing investment increased by 155% (RR = 1.987, 95% CrI: [1.144, 3.458]), indicating suppression, indicating that ADI and segregation initially masked its association with firearm homicide risk.

**Conclusion:**

Findings highlight the need for policies that address the long-term effects of lending discrimination. Reverse redlining—where financial institutions target minority communities with high-cost loans—further exacerbates existing inequities. Additionally, neighborhood deprivation and segregation shape firearm-related violence risk, underscoring the broader consequences of systemic housing discrimination.

**Supplementary Information:**

The online version contains supplementary material available at 10.1186/s40621-025-00579-9.

## Introduction

Firearm-related violence in Chicago, Illinois, has reached epidemic levels, creating a public health crisis. From 2012 to 2023, the city recorded the highest number of gun violence fatalities in the United States. In 2016 alone, over 4300 shootings and more than 760 murders marked a 50% increase from the previous year [46]. These statistics conceal significant racial and geographic inequalities. In 2023, the chances of becoming a victim of firearm-related violence were 1 in 915 across the city, but in Fuller Park, a low-income community of color, the odds climbed to 1 in 79–11.6 times the city average—whereas Beverly, a wealthy, predominantly White area, recorded no shootings (Author calculations). Between 2016 and 2018, Black youth aged 15 to 24 were 35 times more likely to die from firearm-related injuries than similarly situated youth in the U.S. and 13 times more likely to die than non-Black youth in Chicago [24, 73].

The stark racial disparities in firearm-related mortality are rooted in both historical and contemporary policies of racial and economic exclusion. Government-backed initiatives, like historic redlining that intensified racial segregation and recent urban renewal projects that disrupted communities, have systematically deprived low-income communities of color of wealth. This was achieved through discriminatory housing policies that prevent specific groups from accessing homeownership. [28, 52, 62, 78]. Ruth Gilmore’s concept of *organized abandonment* captures this process, emphasizing that economic marginalization is not merely the result of past injustices but an active and intentional system that continuously withdraws resources, services, and opportunities from low-income communities of color, reinforcing their sociospatial exclusion [28]. In cities like Chicago, these systems continue to shape racial segregation, social deprivation, and neighborhood-level health disparities, including disproportionate exposure to firearm-related violence [33, 49].

Research consistently links racial disparities in firearm-related violence to structural factors such as redlining, economic inequality, and concentrated poverty, all of which contribute to residential segregation [6, 39, 56, 67]. However, existing studies have examined historic redlining [7, 20, 38, 56] or present-day housing policies [10, 30, 43, 71] independently, but few have considered both. One exception is a study that found neighborhoods in Baltimore exposed to both historical redlining and contemporary socioeconomic disadvantage experienced the highest rates of non-fatal shootings, explaining over one-third of such shootings between 2015 and 2019 [76]. Moreover, past studies have significantly overlooked the impact of both historical (e.g., redlining) and contemporary (e.g., predatory) lending practices on targeted neighborhoods and their surrounding areas, limiting our understanding of how housing discrimination contributes to spatial contagion [16, 69]. This study addresses these gaps by examining both historical and contemporary lending practices and their relationship to firearm-related homicides in Chicago. Using Bayesian spatial modeling, we investigate how lending trajectories influence the relative risk of firearm-related mortality, while taking into account area-level deprivation, racial segregation, and spatial dependence.

## Reframing firearm-related violence as a structural problem

Social Disorganization Theory (SDT) is frequently used to explain how economic inequality and racial segregation contribute to neighborhood crime and violence by weakening social ties and eroding informal social control mechanisms. According to SDT, a major source of variation in neighborhood violence is attributed to the inability of “neighborhoods to realize the common values of residents and maintain effective social control ([65], p. 918).” Lack of social control undermines collective efficacy, which is defined as “social cohesion among neighbors combined with their willingness to intervene on behalf of the common good” [64, 65, p 918). A key assumption is that residents'willingness to maintain public order can achieve the shared goal of being free from interpersonal violence [65].

SDT attributes neighborhood crime and violence to weakened social ties and ineffective informal social control, disproportionately impacting low-income communities of color. By reducing complex neighborhood conditions to demographic indicators—such as the percentage of female-headed households or the proportion of Black residents—SDT offers surface-level explanations that obscure systemic racism as a fundamental driver of violence. The implication for social policy is that residents of marginalized communities are expected to address problems created by government policies from the outset, or that more formal mechanisms of social control are needed to maintain public safety, such as aggressive policing. In contradistinction, public health approaches emphasize the underlying structural inequities associated with being a victim of firearm-related violence, as well as the "upstream effects of structural racism"([29], p. 2). Structural racism, defined by Bailey et al. [4] as "the totality of ways societies foster racial discrimination through mutually reinforcing systems (Bailey et al., [4], p. 1455),” perpetuates the social and physical neighborhood conditions that contribute to the risk of being a victim of violence [3, 62].

## Housing policy and firearm injury

Government-sponsored housing policies have long reinforced racial segregation by restricting access to credit and investment in minoritized communities [36]. Established in 1933, the Homeowners’ Loan Corporation (HOLC) sought to stabilize housing markets by refinancing home mortgages at risk of foreclosure [1]. However, its neighborhood grading system, known as redlining, explicitly incorporated racial bias, assigning ratings from A ("desirable") to D ("hazardous"). Predominantly Black and immigrant neighborhoods were labeled"hazardous,"leading to widespread disinvestment as banks and lenders denied mortgages in these areas while concentrating investments in White, economically'desirable'communities [51, 59]. For example, between 1940 and 1970, redlined neighborhoods only saw a 16% increase in housing supply alongside population decline, indicating sustained disinvestment while investments in housing and infrastructure were diverted to favored neighborhoods [48, 59].

Consistent with organized abandonment [28], the combined effects of historical and contemporary housing discrimination have contributed to sustained disinvestment and socioeconomic disparities, particularly in communities of color. Despite the passage of the Fair Housing Act over three decades ago, racial disparities in mortgage lending have persisted. Prior to the 2008 financial crisis, subprime lending disproportionately targeted minority borrowers with exploitative terms—a practice known as"reverse redlining"(Rothstein 2017). Faber [25] found that before the 2008 financial crisis, mortgage applicants in D-rated neighborhoods were 69% more likely to be denied a loan and 257% more likely to receive a subprime mortgage than those in A-rated areas [25]. After the recession, foreclosure rates were significantly higher in these historically redlined neighborhoods [25]. Predatory lending, characterized by excessive fees and high interest rates, further exacerbated financial instability and foreclosure risks, perpetuating cycles of neighborhood disinvestment and economic decline [23].

Presently, Black and Latine homebuyers continue to experience disproportionately high loan denial rates and financial losses compared to White applicants [37, 79]. Whereas neighborhood reinvestment strategies via public funding mechanisms such as Community Development Block Grants (CDBG) and Tax Increment Financing (TIF) have stimulated economic growth in some neighborhoods [27, 57], these same strategies have reinforced racial and economic divisions in other areas through gentrification. Gentrification intensifies racial and economic segregation by displacing long-term, low-income residents of color while directing investment toward wealthier, White-majority areas, further widening socioeconomic disparities [40, 44]. This systemic exclusion has created lasting economic disparities to this day, manifesting as slower economic development, lower property values, heightened segregation, and higher rates of subprime lending and foreclosure [9, 53, 55]. Therefore, while understanding the historical context of discriminatory housing policies is essential for addressing contemporary firearm-related violence, it is important to examine how modern lending practices—such as predatory and subprime lending—continue to drive socioeconomic disparities and elevate gun violence risk today.

## The lasting impact of redlining on firearm-related violence

Redlining may increase firearm-related violence both directly and indirectly. Residents of redlined neighborhoods experience higher rates of chronic illness, poverty, and limited access to quality education and employment—factors that contribute to increased violence exposure. Several studies have examined associations between HOLC grading and the spatial distribution of firearm-related violence, finding that D-rated neighborhoods have at least five times more firearm victims than A-rated areas, even after controlling for sociodemographic factors and structural vulnerabilities [7, 12, 20]. White et al. [77] found that individuals in A- and B-rated neighborhoods had significantly higher quality-of-life indicators than those in C- and D-rated areas [77]. Similarly, Diaz et al. [21] found a stepwise decline in 30-day post-surgical survival rates across HOLC-graded neighborhoods, with the highest mortality observed in"hazardous"(D-rated) neighborhoods, even after adjusting for modern-day area deprivation [21]. However, associations between racial and economic segregation and firearm-related injury have yielded mixed results. Knopov et al. [45] found that racial segregation, as measured by the index of dissimilarity, was positively associated with Black-White firearm homicide disparities [45]. Conversely, other studies have found no direct association between racial segregation and firearm homicide rates [15, 42].

## Present study

**Fig. 1:**
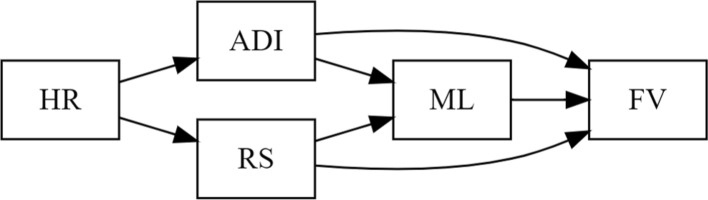
Directed acyclic graph (DAG) illustrating hypothesized relationships among key variables: Historical redlining score (HR), area deprivation (ADI), racial segregation (RS), mortgage lending (ML), and firearm-related violence (FV). Area deprivation (ADI) and racial segregation RS are conceptualized as confounders of the association between mortgage lending (ML) and firearm-related violence (FV), shaped by historic redlining (HR) but not influencing it. Arrows indicate hypothesized pathways. The conceptual model reinforces our theoretical model positing that structural racism manifests through both historical and contemporary policies and practices

Despite emerging research on the long-term effects of redlining on firearm-related violence, a key gap remains in understanding how the combined influence of historical and contemporary housing policies continues to shape gun violence risk in the present day. To address this research gap, we examine how historical and contemporary housing policies are associated with firearm-related violence, with particular attention to the roles of residential racial segregation and area-level deprivation. We conceptualize historical redlining as a fixed, past exposure that shaped long-term patterns of racialized residential segregation and area-level deprivation (ADI; Fig. 1). Because both racial segregation and ADI are measured contemporaneously and are influenced by historical redlining, we do not treat them as confounders. Instead, we view them as downstream mediators through which redlining may exert its influence on present-day firearm violence. However, we hypothesize that ADI and residential segregation may confound the relationship between current mortgage lending discrimination and firearm-related violence. Conceptually, area deprivation and residential segregation may act as confounders in the relationship between contemporary mortgage lending and firearm violence, as both shape neighborhood conditions that influence investment patterns and violence risk within and across redlined areas.

Building on this conceptual framework, we aim to empirically test how historical and contemporary housing policies contribute to present-day disparities in firearm-related violence. Our specific research questions are: (1) What is the relative risk of gun-related homicide in historically redlined neighborhoods that currently experience low lending rates and excessively high-cost loans (indicating ongoing discrimination) compared to neighborhoods with higher lending rates and lower high-cost loan burden? and (2) Do neighborhood deprivation and racial segregation explain the relationship between historical and current lending practices and victimization from firearm-related violence? Based on past research, we expect that the risk of gun-related homicide will be highest in historically redlined neighborhoods with lower mortgage originations and higher loan-to-value ratios presently and that the risk will exert a direct effect beyond present-day deprivation and demographic factors.

## Methodology

### Data

*Home mortgage disclosure act data (HMDA).* We used publicly available data collected under the Home Mortgage Disclosure Act [34] for 2019 (HMDA; Federal Financial Institutions Examination Council 2019), a U.S. federal law mandating banks, credit unions, and mortgage lenders to report detailed annual data on mortgage applications, originations, and purchases to regulatory agencies. The primary purpose of the HMDA is to provide information about the effectiveness by which financial institutions serve their communities'housing needs, including lending practices and the distribution of mortgage loans. Key data points encompass applicant demographics (such as race, ethnicity, and income), loan characteristics (type, purpose, amount), and property location.

*Homeowners’ loan corporation (HOLC) data*. The HOLC created residential security maps, commonly known as"redlining"maps, which categorized neighborhoods based on perceived credit risk. These maps heavily influenced lending practices by financial institutions, discriminating against areas with higher concentrations of minoritized or lower-income residents. We obtained HOLC data from Meier et al. (2021), who integrated HOLC residential security maps from the Mapping Inequality Project [58] with 2010 census tract boundary files. They assigned a numerical value referred to as a historical redlining score (HRS) to each census tract based on HOLC risk categories as follows: “A” (Best) = 1, “B” (Still Desirable) = 2, “C” (Definitely Declining) = 3, “D” (Hazardous) = 4. The HRS was calculated as the weighted average of HOLC grades within its boundaries. Tracts with more than 50% ungraded area were excluded from the analysis, ensuring that scores reflected the actual HOLC designations. The data are publicly available and can be accessed here: https://archive.icpsr.umich.edu/view/studies/141121 (Meier and Mitchell 2023).

*Firearm-related homicides*. We used publicly available data from the Cook County, Illinois, Medical Examiner Case Archives. We included all firearm-related deaths classified as homicides that occurred in Chicago in 2019. We excluded cases that did not have a documented point location (latitude and longitude) of the incident (n = 14) and that occurred in census tracts for which we could not determine lending trajectory (n = 58), resulting in 400 individual reports.

## Measures

Racial diversity was based on Simpson’s Index of Diversity $y=1-\sum_{k}{(\frac{n}{N})}^{2}$ where *n* is the number of residents of a particular group, and *N* is the number of persons in each census tract. The index measures the likelihood that two randomly selected individuals belong to separate racial groups. The index ranges from 0 to 1, with higher values indicating greater diversity and lower values indicating higher segregation. Race was divided into seven census-defined categories: White, Black, Native American, Asian, Pacific Islander, Hispanic, and ‘some other race.’ The index was created using data from the American Community Survey (ACS) 5-year estimates for 2019 [75].

*Area deprivation index*. We used the *get_adi* function from the *sociome* [48] package in R to download the Area Deprivation Index (ADI; [68]) at the census tract level for 2019. The ADI is a composite measure of seventeen indicators ranging from 1 (less deprived) to 100 (most deprived) constructed from weighted factor score coefficients to describe a neighborhood’s relative socioeconomic position across three domains: financial strength, economic hardship and inequality, and educational attainment. We grouped the ADI rankings into deciles where the highest (lowest) decile describes the most (least) deprived 10% of neighborhoods in the city. The most advantaged 10% of neighborhoods served as the reference category for the ADI decile.

*Lending trajectory*. We combined historic redlining scores (HRS) from Meier et al. (2021) with the 2019 HMDA data to create a lending trajectory variable that categorizes lending discrimination into past and present. First, we dichotomized the HRS values at the 25 th percentile, following [54] who used this percentile-based cutoff to differentiate historically redlined areas with the highest levels of mortgage risk from less affected areas. The 25 th percentile cutoff is necessary to identify neighborhoods most impacted by historic lending discrimination and systemic disinvestment while retaining enough variation for meaningful comparisons. Predatory lending refers to neighborhoods where at least 15% of loans originated are deemed high-cost, characterized by a rate spread exceeding 1.5 percentage points above the average prime offer rate (APOR) for a similar type of loan [53]. We defined a loan as high-cost if its rate spread exceeded 1.5 percentage points above the average prime offer rate (APOR), consistent with federal lending regulations updated in 2009. The APOR, published by the Federal Reserve, reflects typical interest rates offered to borrowers with good credit (Center for Responsible Lending 2009). A rate spread greater than 1.5% indicates that the borrower is paying substantially more than the market average, which is one indication of predatory lending. Census tracts were classified as experiencing current lending discrimination if they had low lending activity (defined as falling within the bottom decile of originated loans citywide based on 2019 HMDA data), a high proportion of high-cost loans (≥ 15% of originated loans with a rate spread > 1.5% above the APOR), or both.

As did [53], we combined the dichotomized HLS scores with the lending discrimination variable to create a four-level categorical lending trajectory: tracts with low historic redlining and no current lending discrimination (high investment), tracts with high historic redlining and no current lending discrimination (growing investment), tracts with low historic redlining and current lending discrimination (disinvested), and tracts with high historic redlining and current lending discrimination (sustained disinvestment). We used disinvested neighborhoods as the reference category in the statistical models.

## Statistical approach

The expected counts represent the population-adjusted number of firearm-related homicides in each census tract. These were derived using population denominators from each tract, consistent with methods commonly used in spatial epidemiology and disease mapping (Li et al. 2019). The number of incidents, ${y}_{i}$ in census tract *i* was assumed to follow a Poisson distribution with mean ${\lambda }_{i}$:

$${y}_{i}\sim Poisson\left({\lambda }_{i}\right),$$

To account for differences in population size across tracts, the logarithm of the expected count (based on population) was included as an additive offset in the linear predictor, resulting in a multiplicative adjustment to the expected counts on the original scale:

$$\text{log}\left({\lambda }_{i}\right)={\text{log}\left({E}_{i}\right)+x}_{i}^{T}\beta +{\alpha }_{i},$$

where ${E}_{i}$ is the expected count based on the population, ${x}_{i}^{T}$ is the vector of covariates for census tract $i$*,*$\beta$ is a vector of fixed effect regression parameters and ${\alpha }_{i}$ is a spatially structured random effect that follows the Besag, York, and Mollie (BYM) model [8]. This model estimates the relative risk of firearm-related homicide while adjusting for spatial dependence and tract-level covariates. The random effect term accounts for unobserved heterogeneity and spatial dependence by incorporating an intrinsic conditional autoregressive (CAR) component and an independent location-specific error term. This approach generated posterior distributions of model parameters and estimated firearm-related homicide counts based on observed data. We used default priors, specifying fixed effects with an improper flat normal prior and a precision parameter to ensure smaller variance components, thereby preventing overfitting while preserving model flexibility. Finally, we computed posterior median risk and exceedance probabilities, which quantify the likelihood that relative risk in a given tract is equal to or exceeds twofold, threefold, or fourfold the citywide average risk for Chicago in 2019.

*Model selection and assumption checks*. The hierarchical Bayesian model was estimated using Integrated Nested Laplace Approximation (INLA) in the R-INLA package [63, 66]. The model selection followed a stepwise approach. Model 1 was fit as a baseline Poisson model without spatial heterogeneity. Model 2 added a spatially structured CAR term. Model 3 incorporated the categorical lending trajectory variable into Model 2. Model 4 added area-level deprivation (deciles) to Model 2. The final model (Model 5) included an intercept, area-level deprivation decile, lending trajectory, racial segregation, and a spatially structured CAR term. The Deviance Information Criterion (DIC), Watanabe-Akaike Information Criterion (WAIC), and the number of effective parameters guided model selection, with lower values indicating a better fit. Model assumptions were assessed via overdispersion tests, posterior predictive checks, and sensitivity analyses of prior distributions. Before finalizing the model, we calculated the variance-to-mean ratio $\left(\frac{{\sigma }^{2}}{\mu }\right)$ using the raw count data to determine whether a Poisson model would be appropriate. To address overdispersion, we incorporated a spatially structured random effect term using the BYM model, which accounts for spatial dependence and unstructured variability, and evaluated models with alternative distributions.

*Model calibration and sensitivity analyses*. We assessed convergence and approximation accuracy through multiple diagnostics to ensure model validity. We examined posterior distributions of fixed effects, ensuring they were smooth and unimodal without excessive autocorrelation. We also checked effective sample sizes (ESS > 200) and posterior standard deviations of hyperparameters, confirming stable parameter estimation. Model calibration was assessed using Probability Integral Transform (PIT) values. We examined alternative prior specifications for regression coefficients, assessed unstructured *iid* random effects, and conducted posterior predictive checks comparing observed and predicted firearm-related homicide counts.

All analyses were conducted using R version 4.3.2 [60].

## Results

*Descriptive analysis of firearm-related homicide.* Table 1 presents descriptive characteristics of the four hundred firearm-related homicide victims in 2019, along with demographic, economic, and social factors, as well as lending practices in their neighborhoods. The average age of victims was 29.09 years, with the majority being male (90.5%). Racial disparities were stark: 81.8% of victims were Black, followed by 17.8% White. Most victims of firearm-related violence within Chicago also resided there (83.8%). Victims of firearm-related violence lived in areas of sustained disinvestment (64.2%), with a smaller proportion in areas experiencing growing investment (25.7%) and only 2.3% in high-investment areas.

**Table 1:** Descriptive characteristics of firearm-related homicide victims and census tracts -- Chicago, IL, 2019

**Victims (n)**	400
** Age, years (mean (SD)) **	29.09 (10.52)
** Gender**	
Female	38 (9.50)
Male	362 (90.50)
** Race **	
Am. Indian	1 (0.25)
Black	327 (81.75)
Other	1 (0.25)
White	71 (17.75)
** Ethnicity **	
Latine	57 (14.25)
Non-Latine	343 (85.75)
** Chicago residence**	335 (83.75)
**Census tracts (n)**	218
** Lending practice (mean (SD))**	
Historic redlining score	3.27 (0.55)
Rate spread	1.43 (1.66)
Loans per 1000	25.48 (27.53)
Property to loan ratio	1.47 (2.56)
** Redlining trajectory**	
Disinvested	17 (7.80)
Growing investment	56 (25.69)
High investment	5 (2.29)
Sustained disinvestment	140 (64.22)
** Neighborhood characteristics (mean (SD))**	
Poverty rate	29.05 (13.01)
Housing age	69.40 (12.55)
Area deprivation index	117.09 (15.54)

Numbers are No. (%) unless otherwise noted. SD = standard deviation.

**Fig. 2:**
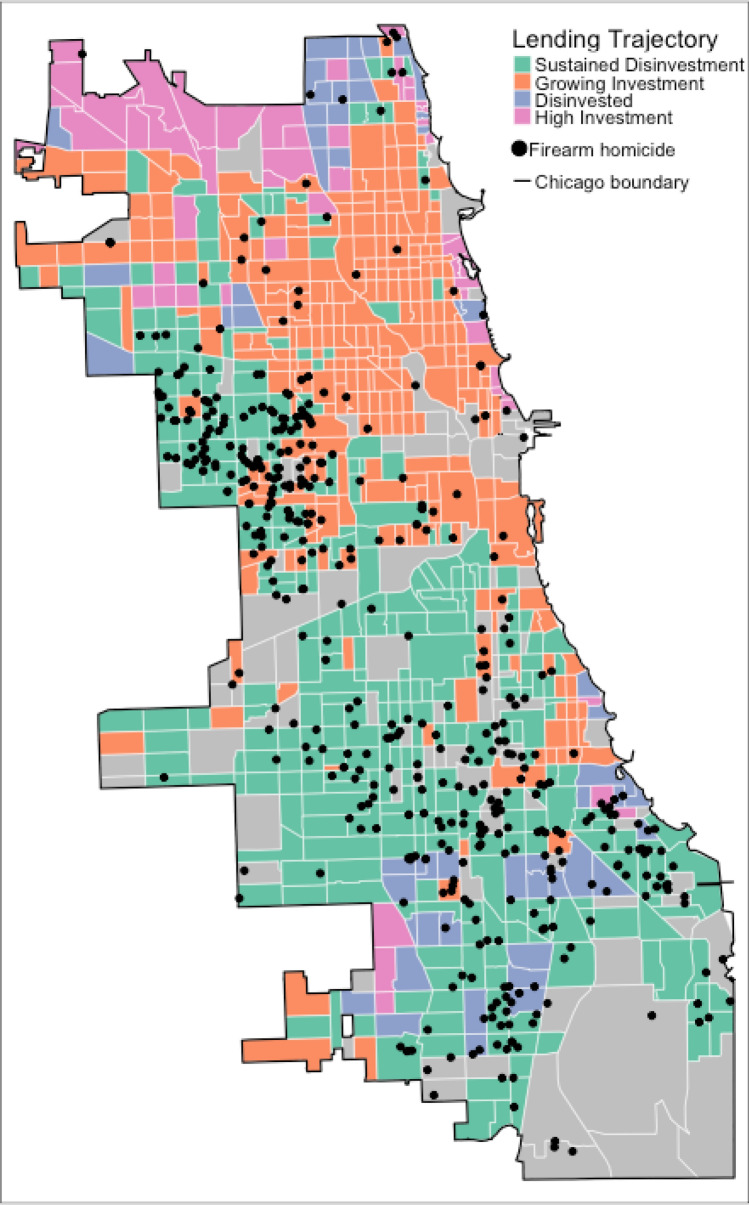
Spatial distribution of firearm-related homicide in Chicago. Map of lending trajectories with point locations of 2019 firearm homicides. Grey census tracts are missing lending trajectory (and were excluded from analyses)

**Fig. 3:**
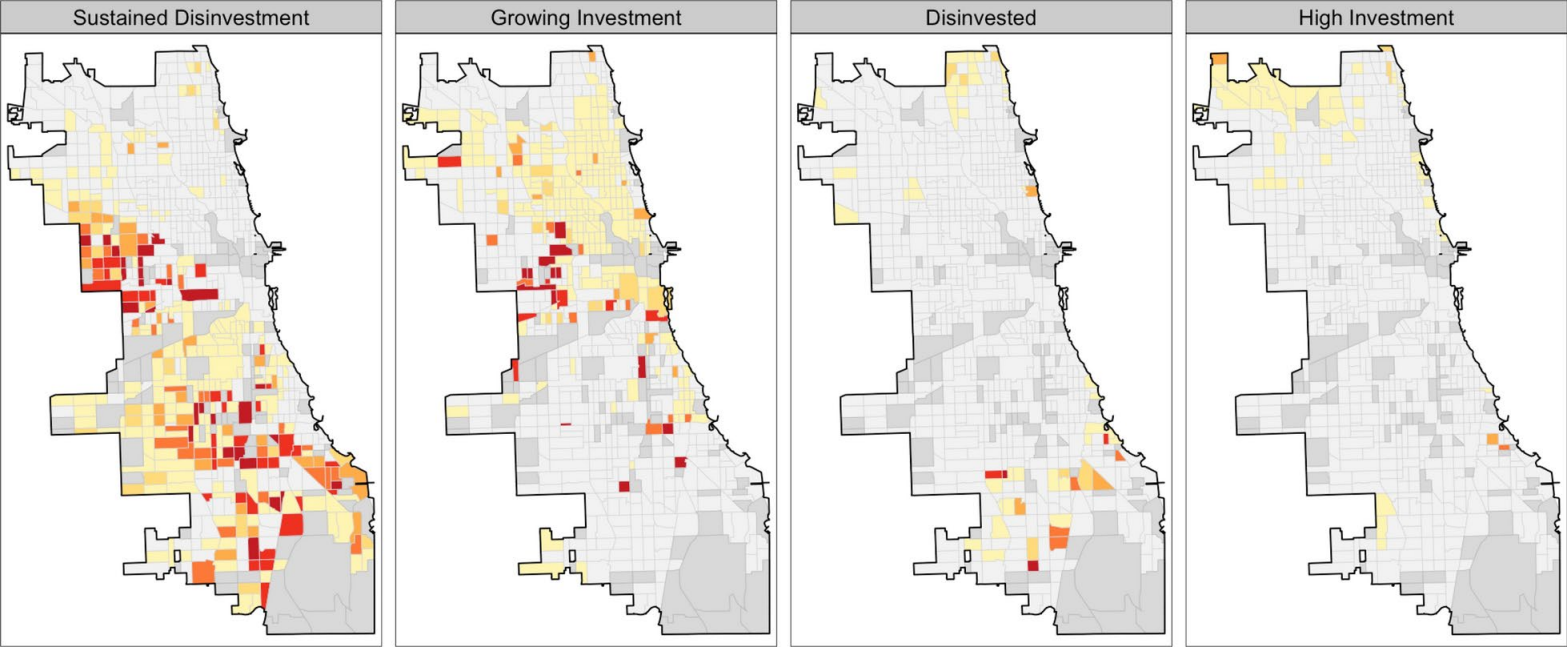
*Conditional map of firearm homicide Standardized Mortality Ratios (SMRs) by lending trajec*tory. Same color palette as previous plot (lightest yellow is no firearm homicides for 2019 (SMR = 0); dark red is highest quantile of SMR; dark grey census tracts are missing lending trajectory and were excluded from analyses)

**Fig. 4:**
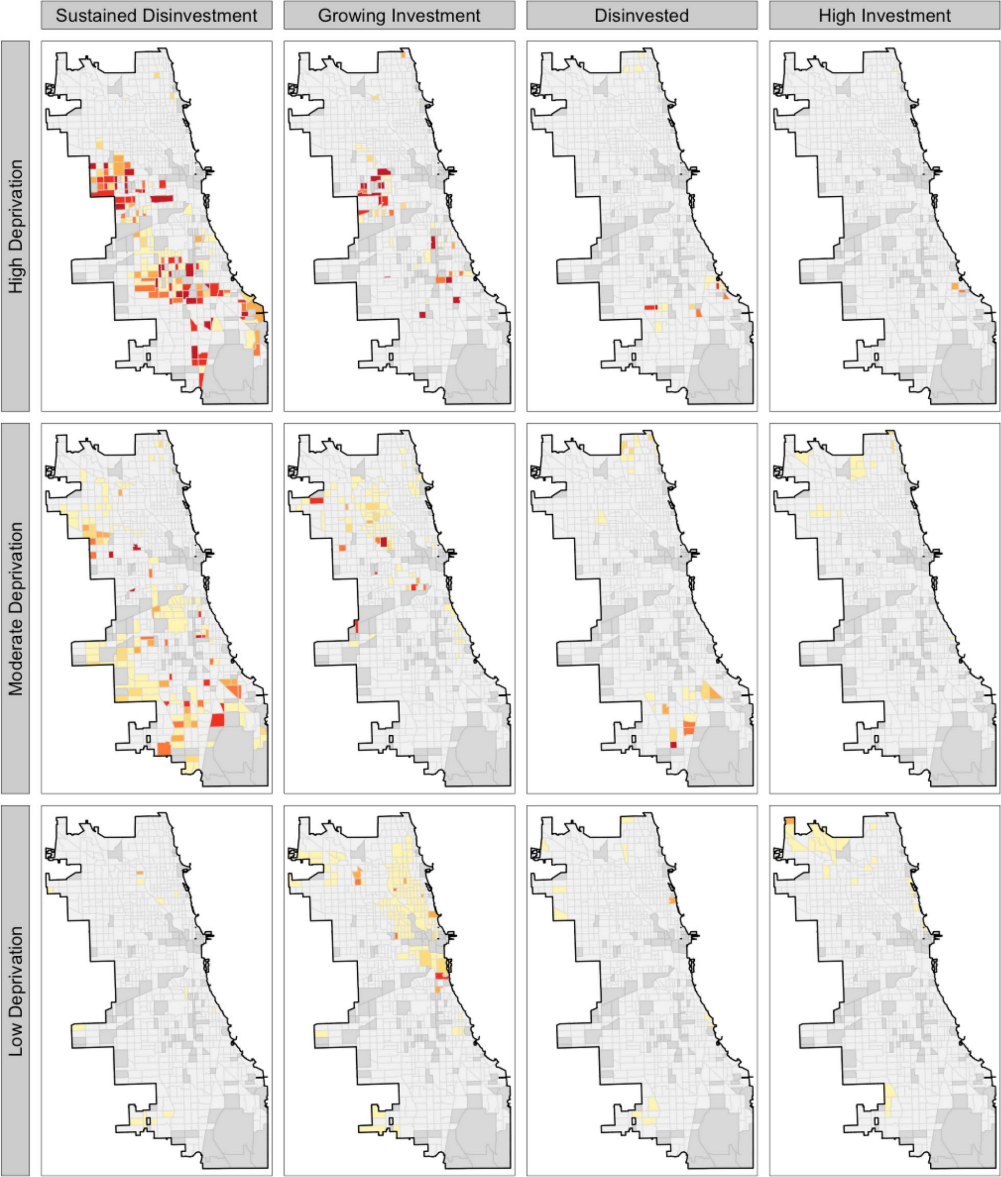
Conditional map of firearm homicide Standardized Mortality Ratios (SMRs) by lending trajectory and Area Level Deprivation (ADI) tertile. Same color palette as previous plots (lightest yellow is no firearm homicides for 2019 (SMR = 0); dark red is highest quantile of SMR; dark grey census tracts are missing lending trajectory and were excluded from analyses)

*Geographic patterns of firearm-related victimization*. Figure 2 confirms that the highest concentration of gun violence appears in areas of sustained disinvestment and growing investment, while neighborhoods with high investment exhibit significantly lower firearm-related homicide rates. Figure 3 presents a conditional map of the Standardized Mortality Ratio (SMR) across lending trajectories, showing a strong spatial association between firearm-related homicide and neighborhood lending patterns. The highest SMRs are concentrated in sustained disinvestment and growing investment neighborhoods, whereas areas of high investment experience markedly lower firearm-related mortality risk. Figure 4 illustrates the bivariate distribution of firearm-related homicide risk across quartiles of the ADI (*y*-axis) and lending trajectory categories (*x*-axis). The results demonstrate that ADI strongly conditions firearm homicide risk, particularly in neighborhoods experiencing sustained disinvestment and growing investment. Figure 4 shows that firearm-related violence is most concentrated in neighborhoods experiencing both sustained disinvestment and high deprivation. Even in areas with growing investment, violence remains more prevalent where deprivation is still high, suggesting that reinvestment alone may not immediately reduce risk. In contrast, neighborhoods with high investment and low deprivation show the fewest incidents, highlighting the protective role of structural advantage.

*Model fit assessments and sensitivity checks.* Table 2 presents the model fit statistics, with Model 5 selected as the best-fitting model due to its lowest DIC (1200.433) and WAIC (1219.30), indicating optimal fit and parsimony. Model 5 also had the fewest effective parameters (91.61), suggesting efficient data representation without overfitting. To assess overdispersion, we calculated the variance-to-mean ratio of firearm-related violence counts ($\frac{{\sigma }^{2}}{\mu }=2.04$), suggesting that a standard Poisson model might not fully capture variability in the dependent variable. We then compared the Poisson and Negative Binomial models; however, the Poisson model demonstrated a better fit (DIC = 1200.433 vs. 1214.501 for the Negative Binomial); therefore, the additional dispersion parameter did not improve performance. Posterior hyperparameter estimates further supported the Poisson model, with minimal variance in the spatially structured random effect $\left(\frac{1}{{\tau }_{u}}=0.0018\right)$. Given that the BYM Poisson model effectively captured spatial dependencies and accounted for overdispersion, it was retained for further analysis.

**Table 2:** Model fit

	DIC	WAIC	Eff.Par
Model 1: Unstructured heterogeneity	1419.26	1479.17	251.55
Model 2: Structured heterogeneity	1347.35	1390.25	206.50
Model 3: Model 2 + redlining category	1290.13	1326.09	172.96
Model 4: Model 2 + area deprivation	1208.31	1225.07	99.46
Model 5: Model 2 + area deprivation + racial segregation	1200.57	1219.30	91.61

Following selection, model calibration assessments confirmed the model's stability and predictive accuracy. The Probability Integral Transform (PIT) values were approximately uniform between 0.20 and 0.80, indicating well-calibrated predictions. However, we noted some deviations at the extremes (PIT ≈ 0 or 1), which suggested localized areas of underprediction and overprediction. Model robustness was further validated through posterior predictive checks, where observed and predicted values aligned closely (MAE = 0.87, RMSE = 1.38). A scatter plot confirmed that predictions closely followed the expected 1:1 relationship, validating model calibration (See Supplementary Appendix Fig. 1). The posterior distributions of fixed effects had low standard deviations (< 0.75), indicating reliable estimates. We further computed Kullback–Leibler Divergence (KLD) values, which were minimal, confirming that prior influence on posterior estimates was negligible. To assess robustness, we conducted a sensitivity analysis by testing weaker priors that allowed for more variability in parameter estimates (*N*(0,10) for β and Γ(0.1, 0.01) for τ). The model with default priors was favored due to its slightly lower DIC (1200.43 vs. 1256.07), which indicated better model fit and demonstrated that the results were robust to alternative prior specifications for model parameters.

*Bayesian model results.* The primary exposure of interest is the lending trajectory presented in Table 3. At the same time, ADI and racial segregation are conceptualized as structural conditions that shape neighborhood risk, potentially confounding the association between contemporary lending patterns and firearm-related homicide. Table 3 compares the baseline (Model 2) and best-fitting (Model 5) Poisson models to assess how adjusting for ADI and racial segregation influences the association between lending trajectories and firearm-related violence. In the baseline model, sustained disinvestment (RR = 2.23, 95% CrI: [1.353, 3.682]) more than doubled the risk of firearm-related violence compared to disinvested areas. In contrast, high investment (RR = 0.146, 95% CrI: [0.054, 0.397]) was associated with a more than sixfold decrease (1/0.146) in the risk of firearm-related violence. No meaningful differences emerged between areas of growing investment and disinvestment since the credible interval includes 1 (RR = 0.782, 95% CrI: [0.452, 1.349]).

**Table 3:** Summary of exponentiated fixed effects

	Model without ADI & racial segregation (Mean [95% CrI])	Model with ADI & racial segregation (Mean [95% CrI])
(Intercept)	0.432 [0.265, 0.698]	0.038 [0.011, 0.124]
**Lending trajectory**		
β_1_: Growing investment	0.782 [0.452, 1.349]	1.987 [1.144, 3.458]
β_2_: High investment	0.146 [0.054, 0.397]	1.031 [0.382, 2.770]
β_3_: Sustained disinvestment	2.230 [1.352, 3.681]	1.714 [1.054, 2.791]
**Area level deprivation**		
β_4_: Q2		1.381 [0.459, 4.154]
β_5_: Q3		0.572 [0.142, 2.307]
β_6_: Q4		4.103 [0.244, 2.863]
β_7_: Q5		4.380 [1.683, 11.403]
β_8_: Q6		6.251 [2.523, 15.490]
β_9_: Q7		9.649 [4.018, 23.188]
β_10_: Q8		12.521 [0.314, 5.204]
β_11_: Q9		14.710 [6.187, 34.962]
β_12_: Q10		18.248 [7.726, 43.089]
β_13_: Racial segregation		0.072 [0.042, 0.124]

The reference category for ADI is Q1 = the least deprived 10% of neighborhoods; the reference category for Lending Trajectory is Disinvestment. *CrI* Credible Interval.

In the adjusted model, which includes ADI and racial segregation as confounders, sustained disinvestment decreased firearm-related violence risk by 23.3% (RR = 1.714, 95% CrI: [1.054, 2.79]) compared to the unadjusted estimate (β = RR.230, 95% CrI: [1.352, 3.681]). This attenuation suggests that ADI and racial segregation account for part of the association between lending trajectory and firearm-related homicide. However, the coefficient on growing and high investment increased in the adjusted model, with the former statistically significant. Specifically, the coefficient pertaining to growing investment *increased* by 155% (RR = 1.99, 95% CrI: [1.145, 3.46]), and areas associated with growing investment have a two-fold increased risk of firearm-related violence after accounting for the effect of ADI and racial segregation. This suggests that failing to account for area-level deprivation and racial segregation may underestimate the true risk associated with growing investment neighborhoods.

The association between area deprivation and firearm-related homicide followed a graded pattern after accounting for the effect of lending trajectory and racial segregation, with the most deprived 10% of neighborhoods demonstrating higher homicide risk than the next 10%, and so forth. The risk becomes particularly pronounced when ADI exceeds the median (Q5 and above). Individuals in the most disadvantaged 10% of neighborhoods (Q10) are 18.25 times more likely to experience gun violence than those in the least disadvantaged 10% (Q1). Racial segregation was also a significant predictor of firearm homicide risk after adjusting for ADI and lending trajectory. The coefficient (RR = 0.07, 95% CrI: [0.04, 0.14]) suggests that more diverse areas are associated with less risk of firearm-related violence.

**Fig. 5:**
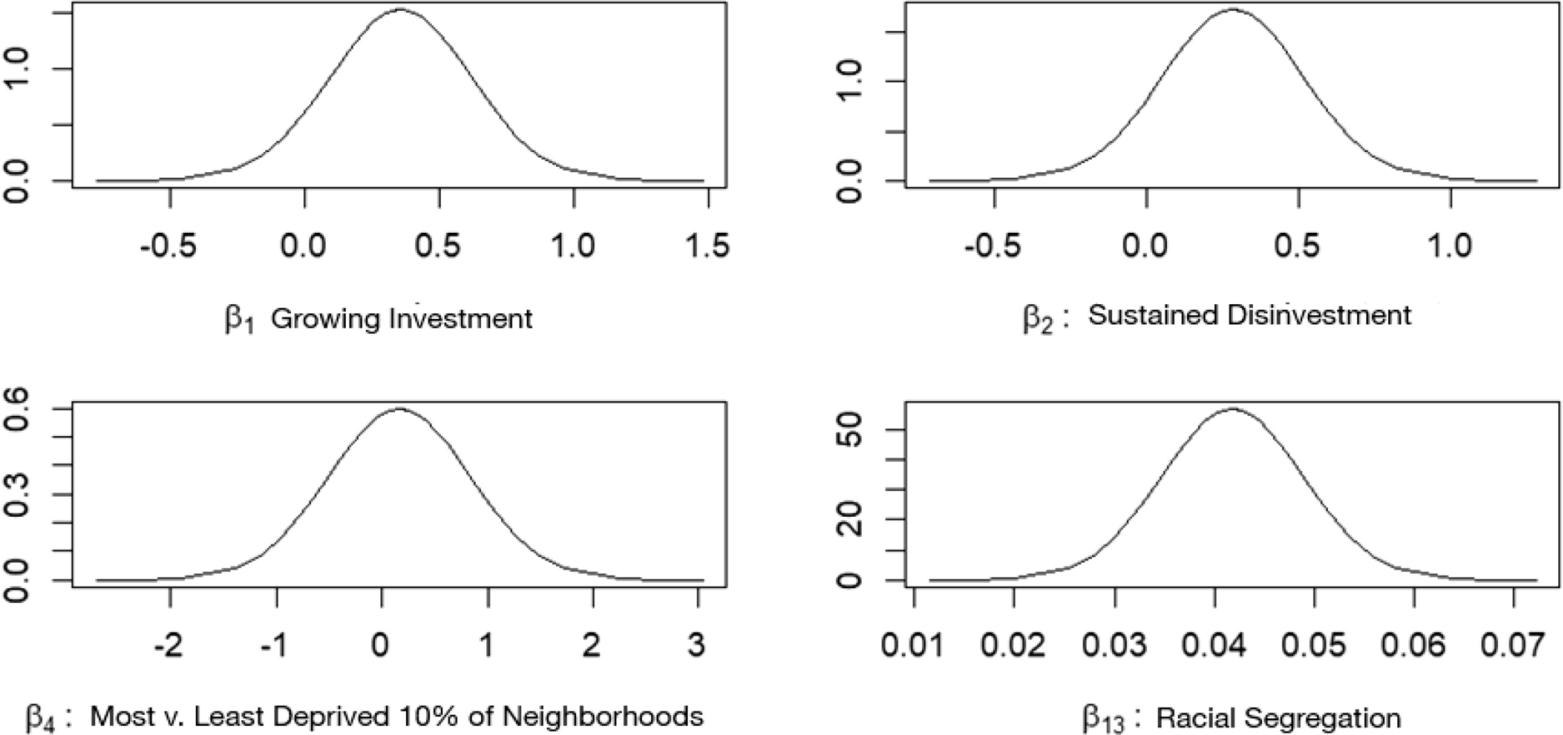
Distribution of beta coefficients and uncertainty estimates. Beta coefficients from the Bayesian spatial model showing the estimates and credible intervals of gun violence relative risk in areas of growing investment, sustained disinvestment, the least deprived 10% of neighborhoods (vs the most deprived 10%), and racial segregation

**Fig. 6:**
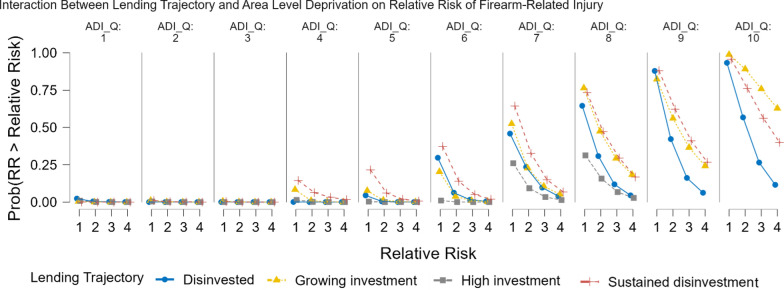
Firearm-related relative risk estimates within Area Deprivation Deciles. Association between firearm-related homicide relative risk (RR > 1, 2, 3 and 4) and Area Level Deprivation (decile) across lending trajectories. The y-axis quantifies the probability that the RR of gun violence is > 1,2,3, or 4 for each lending trajectory within ADI deciles

Posterior parameter estimates are visualized in Fig. 5, illustrating the relative influence of lending trajectory, area deprivation, and racial segregation on firearm-related homicide risk. We also calculated the probability of firearm-related homicide risk exceeding one-, two-, three-, and fourfold the citywide average across lending trajectories and ADI deciles (Fig. 6). The results show that firearm-related homicide was near zero in the least deprived 30% of neighborhoods across all lending trajectories. In contrast, firearm-related homicide risk was heavily concentrated in the most deprived 20% of neighborhoods. The probability of a fourfold increase in firearm homicide risk in the most deprived 10% of neighborhoods was 0.72, 0.49, and 0.23 for growing investment, sustained disinvestment, and disinvestment areas, respectively. Notably, no neighborhoods in the most deprived 20% had transitioned into high-investment areas. Also, in the most deprived neighborhoods, areas of growing investment had the highest relative risk of firearm-related homicide. However, in other disadvantaged areas, sustained disinvestment neighborhoods exhibited the highest firearm homicide risk.

To further contextualize the results, Table 4 compares firearm-related homicide, socioeconomic conditions, and lending characteristics across distinct levels of investment in census tracts. Areas experiencing sustained disinvestment exhibited the highest standardized mortality ratio (SMR = 1.98), the highest historic redlining scores (3.42), the greatest area deprivation (115.65), and the highest levels of residential racial segregation (0.35). These areas also had the lowest loan availability, with only 24.24 loans per 1,000 residents. Neighborhoods characterized by growing investment had the second-highest SMR (0.81), similar historic redlining scores (3.40), and slightly lower residential racial segregation (0.34). However, these areas showed greater access to financial resources, with significantly higher loan availability (63.49 per 1,000 residents), fewer high-cost loan originations (0.50), and lower area deprivation (93.53) compared to sustained disinvestment neighborhoods.

**Table 4:** Area Level Characteristics within Lending Trajectories

Variable	Overall	Sustained disinvestment	Disinvested	Growing investment	High investment	*p *value
*# *of Census tracts	721	317	58	292	54	
Standardized mortality ratio	1.277 (2.656)	1.968 (3.124)	0.817 (1.628)	0.821 (2.298)	0.179 (0.607)	<0.001
Probability of RR > 4	0.090 (0.204)	0.145 (0.235)	0.021 (0.042)	0.060 (0.190)	0.002 (0.013)	<0.001
Relative risk	1.352 (1.736)	2.047 (1.784)	0.839 (0.909)	0.912 (1.671)	0.197 (0.363)	<0.001
Historic redlining score	3.241 (0.608)	3.425 (0.468)	2.326 (0.325)	3.401 (0.469)	2.277 (0.434)	<0.001
Loans per 1000	41.749 (35.278)	24.243 (25.524)	27.034 (17.960)	63.493 (37.244)	42.741 (19.598)	<0.001
Rate spread	1.338 (1.645)	1.987 (1.732)	2.483 (2.113)	0.500 (0.951)	0.833 (1.023)	<0.001
% Minoritized Pop.	68.639 (30.125)	87.223 (18.909)	71.364 (28.572)	53.349 (28.738)	39.293 (23.342)	<0.001
Racial segregation	0.389 (0.214)	0.353 (0.268)	0.437 (0.173)	0.342 (0.231)	0.441 (0.185)	<0.001
Population (2019)	3,495.394 (1809.748)	3,429.927 (1693.641)	4,359.897 (1723.399)	3,183.421 (1856.054)	4,638.130 (1603.666)	<0.001
Housing age	67.105 (13.812)	68.606 (10.924)	67.362 (8.041)	65.613 (17.608)	66.093 (9.014)	0.058
Property to loan ratio	1.216 (1.957)	1.244 (1.785)	1.664 (3.780)	1.123 (1.777)	1.075 (0.163)	0.255
Interest rate	4.711 (0.602)	4.908 (0.692)	4.710 (0.741)	4.516 (0.429)	4.615 (0.142)	<0.001
Area deprivation	104.001 (20.067)	115.651 (13.512)	105.577 (14.720)	93.533 (20.558)	90.531 (15.018)	<0.001

Numbers are No. (%) unless otherwise noted. *SD* standard deviation, *IQR * interquartile range

## Discussion

This study examined the relationship between historical and contemporary housing discrimination and the risk of firearm-related victimization while considering the broader neighborhood contexts of racial segregation and area-level deprivation. Like others before us, we found that firearm-related violence is disproportionately concentrated in racially and economically marginalized neighborhoods [18, 22] with young Black males bearing the highest burden [17, 45]. Notably, eight in ten victims were Black, reinforcing the urgent need to reframe firearm-related violence as not only a public health crisis but also a civil rights issue.

Firearm-related violence was spatially concentrated in only 218 of Chicago’s 866 census tracts (25%). Most victims were shot in areas of sustained disinvestment (64.22%), followed by growing investment (25.69%), while only 2.29% were shot in high-investment neighborhoods. Victimization was disproportionately concentrated in areas characterized by financial instability, economic inequality, and low educational attainment, reinforcing prior research linking structural disadvantage to firearm-related violence [22, 32, 74]. Firearm-related homicide risk followed a stepwise gradient, increasing as neighborhood deprivation became more entrenched, with the most socioeconomically disadvantaged 20% of neighborhoods experiencing the highest rates.

Consistent with prior research, we found that firearm-related violence is highest in neighborhoods shaped by historical and ongoing economic exclusion due to redlining [14, 31, 70]). Our findings indicate that firearm-related violence was highest in areas with both a history of redlining and ongoing discriminatory lending practices after accounting for the effects of ADI and racial segregation. After adjusting for ADI and racial segregation, the association between sustained disinvestment and firearm-related homicide decreased by 23.3%, suggesting that (1) present-day disinvestment contributes to firearm-related violence and (2) part of the relationship between sustained disinvestment and firearm-related homicide operates through neighborhood deprivation and segregation, rather than being driven solely by investment patterns.

Our findings further highlight how the legacy of redlining continues to shape firearm-related violence, particularly in neighborhoods classified as areas of growing investment and sustained disinvestment [2, 5, 19]. Both types of neighborhoods were historically redlined, yet sustained disinvestment areas remain subject to ongoing discriminatory lending practices, resulting in continued economic exclusion. Past research has identified homeownership as a protective factor against firearm-related violence because it strengthens community ties and provides residents with greater access to resources [50, 59]. However, our results question whether investment in homeownership alone is sufficient to reduce firearm-related violence—particularly when investment does not address deeply entrenched racial and socioeconomic disparities resulting from decades of systemic disinvestment. Together, our findings reinforce the argument that firearm-related violence stems from organized abandonment [28], where long-term economic withdrawal from marginalized communities fuels disparities in violence exposure.

Discriminatory lending practices have systematically excluded communities of color from wealth-building opportunities, contributing to economic instability and intergenerational wealth loss [37]. Our findings reinforce prior research demonstrating that, even after redlining was officially outlawed, discriminatory housing policies persist as structural determinants of firearm-related homicide [11]. While the Fair Housing Act was intended to prevent racial discrimination in lending, racial disparities in mortgage approvals and loan terms persist, shaping ongoing patterns of economic exclusion, housing instability, and firearm-related violence. Rather than reducing violence, reinvesting in historically disinvested neighborhoods may exacerbate socioeconomic instability if the investment is not equitably distributed. This aligns with research showing that reinvestment can reinforce spatial and economic inequalities without equity-focused policies, leading to social fragmentation and displacement [48, 72]. Studies finding that neighborhood revitalization efforts, such as demolishing vacant and deteriorating properties, have had limited impact on reducing violent crime are also consistent with our results [41]. In contrast, some studies demonstrate that targeted community investment initiatives—such as increasing tree canopy coverage [61], implementing trauma-informed approaches in schools [6] improving labor market outcomes [13], and reducing housing instability [43], Stansfield and Semenza, 2023– can improve safety when paired with policies that target structural inequities.

In this study, firearm-related violence risk was highest in growing investment neighborhoods located in the most socioeconomically deprived 10% of census tracts, underscoring the role of neighborhood deprivation in areas experiencing economic revitalization. Current reinvestment strategies often fail to mitigate the long-term consequences of systemic disinvestment when they prioritize less disadvantaged areas over those with greater socioeconomic vulnerability [44, 48, 57]. On this basis, Burrowes (2020) argues that revitalizing historically excluded Black neighborhoods requires a racial equity framework—one that aligns economic development with the needs of long-term residents. Without targeted protections for vulnerable residents, reinvestment may accelerate displacement, weaken social cohesion, and ultimately exacerbate firearm violence [48]. In line with our critique of SDT, a more nuanced analysis of the structural conditions that shape collective efficacy and social cohesion provides deeper insight into why some communities experience weakened informal social control, whereas others do not. This perspective also informs violence prevention efforts, as seen in the present case, where policies ensure that residents of neighborhoods experiencing sustained disinvestment or growing investment have access to mechanisms that support wealth generation, including homeownership.

Given that ADI and racial segregation attenuate the effect of sustained disinvestment on firearm-related violence, a key methodological consideration is whether these variables should be included as covariates in the Bayesian spatial model or conceptualized as mediators (Edwards et al. 2024). If the goal is to disentangle these pathways, including ADI and racial segregation in the model offer a clearer interpretation of the direct effect of lending trajectory while accounting for its indirect influence through structural disadvantage. Surprisingly, our findings indicate that ADI and racial segregation function as both mediators and suppressors, depending on the lending trajectory. Sustained disinvestment contributes to firearm-related violence primarily through its effects on neighborhood deprivation and segregation, reinforcing their role as mediators. However, for growing investment, ADI and racial segregation functioned as suppressors—their inclusion in the model revealed a stronger association between growing investment and firearm-related violence. Considering both results, retaining ADI and racial segregation as control variables in the models was important for several reasons. First, the attenuation of sustained disinvestment was only partial, meaning ADI and racial segregation explain some, but not all, of its association with firearm violence. Second, adjusting for ADI and racial segregation helps isolate the association between contemporary mortgage lending discrimination and firearm violence by accounting for potential confounders of this specific relationship. Third, the 155% increase in the effect of growing investment after adjustment suggests that failing to account for neighborhood deprivation and segregation obscured its true relationship with firearm-related violence. Finally, excluding ADI and racial segregation prevents disentangling direct and indirect effects, limiting interpretability. Future research would benefit from examining the interrelationships between these variables using spatial mediation models to quantify these indirect effects further and refine the causal pathways linking historical and contemporary housing discrimination to firearm-related violence.

## Limitations, strengths, and directions for future research

We incorporated an innovative framework, proposed by Lynch et al. [54], to investigate trajectories of historical and current housing discrimination and examine the relative risk of firearm-related violence using Bayesian spatial modeling. The study's strengths include the incorporation of spatial dependence and the merging of multiple large datasets, including the most comprehensive data on loan originations available. Nevertheless, our study is not without limitations. First, Chicago is one of the most violent cities in the United States, and therefore, our results may not be generalizable to other contexts. The unique context in Chicago only underscores the need for more research to continue to examine the impact of government-sponsored discrimination and private lending practices on contemporaneous gun violence. Also, we focused on firearm-related mortality rather than firearm-related injuries that did not result in death. As well, we focused on the cause of death, i.e., a gunshot wound, rather than the manner of death. The associations we found may differ by manner of death, including suicide and accidents. Specifically, the relative risk of suicidal injury or death from a self-inflicted gunshot might be higher in areas of high investment, which are often linked to White neighborhoods. However, research has increasingly shown that suicidal injury risk is associated with socioeconomic deprivation [26] and declines in community investment, including home values and tax revenue [35]. Our measure of ADI comprises seventeen indicators that measure socioeconomic deprivation. However, there may be omitted variables that we did not account for in our analysis. Furthermore, conditioning on ADI and residential segregation may introduce collider bias if these variables mediate the relationship between historic redlining and firearm-related violence, particularly when confounders of the mediator-outcome relationship are not accounted for. In this regard, this study is observational, and the identified associations should not be viewed as causal. Future research would benefit from replicating these results using quasi-experimental methods or causal mediation models. Finally, our measure of current discrimination may yield different findings if high-cost loans and low lending rates were separated. Future research should continue to examine the public health impact of discriminatory housing policies in U.S. cities and other social contexts.

### Conclusion

Addressing gun violence in Chicago will require not only targeted interventions to reduce immediate risks but also long-term strategies to dismantle the systemic inequities that underlie these patterns of violence. Future research should continue to explore the intersection of housing policy, economic deprivation, and firearm violence, particularly in urban environments with histories of racial and economic segregation.

## Supplementary Information

Additional file1

## Data Availability

All data used in this manuscript are publicly available. Homeowners'Loan Corporation data used in this study are available from the Harvard Dataverse located here https://www.openicpsr.org/openicpsr/project/141121/version/V2/view?path =/openicpsr/141121/fcr:versions/V2/HRS2020-Shapefiles&type = folder. Home Mortgage Disclosure Act data is provided by the Federal Financial Institutions Examination Council (FFIEC) and can be accessed here https://ffiec.cfpb.gov/. American Community Survey Estimates can be accessed from the United States Census Bureau located here https://www.census.gov/programs-surveys/acs/data.html. Data on firearm-related violence is available at the Cook County Medical Examiner's Case Archive Open Data Portal located here https://datacatalog.cookcountyil.gov/.

## Abbreviations

HOLC: Homeowners’ Loan Corporation
HMDA: Home Mortgage Disclosure Act
ADI: Area Deprivation Index
SDT: Social Disorganization Theory
BYM: Besag, York, and Mollie (a model used in spatial statistics)
INLA: Integrated Nested Laplace Approximation
ACS: American Community Survey
SMR: Standardized Mortality Ratio
CrI: Credible Interval
APOR: Average Prime Offer Rate
CDBG: Community Development Block Grants
TIF: Tax Increment Financing
