# Supplementary material for: Redlining, reinvestment, and racial segregation: a bayesian spatial analysis of mortgage lending trajectories and firearm-related violence
Source: Inj Epidemiol. 2025 May 2;12:23. doi: 10.1186/s40621-025-00579-9 (PMC12046721; doi:10.1186/s40621-025-00579-9)
Supplement: Supplementary file 1 — Additional file1 [file 40621_2025_579_MOESM1_ESM.docx]

**Supplementary Appendix**

**SQL code used to select variables from HMDA data for 2019**

DROP TABLE IF EXISTS public.il2019F1;

CREATE TABLE il2019F1 AS (SELECT

lar_2019.activity_year,

lar_2019.derived_msa_md,

lar_2019.state_code,

lar_2019.county_code,

lar_2019.census_tract,

lar_2019.debt_to_income_ratio,

lar_2019.combined_loan_to_value_ratio,

lar_2019.income,

lar_2019.property_value,

lar_2019.loan_amount,

lar_2019.interest_rate,

lar_2019.applicant_ethnicity_1,

lar_2019.applicant_race_1,

lar_2019.denial_reason_1,

lar_2019.tract_minority_population_percent,

lar_2019.tract_to_msa_income_percentage,

lar_2019.tract_population,

lar_2019.tract_owner_occupied_units,

lar_2019.tract_median_age_of_housing_units,

lar_2019.rate_spread,

lar_2019.hoepa_status,

lar_2019.lien_status

FROM

public.lar_2019

WHERE lar_2019.loan_purpose = '1' and

lar_2019.action_taken = '1' and

lar_2019.loan_type = '1' and

lar_2019.derived_dwelling_category = 'Single Family (1-4 Units):Site-Built' and

lar_2019.occupancy_type = '1' and

lar_2019.state_code = 'IL' and

lar_2019.county_code = '17031' and

(lar_2019.property_value) != 'NA' and (lar_2019.property_value) != 'Exempt' and

(lar_2019.income) != 'NA' and

(lar_2019.loan_amount) != 'NA' and lar_2019.income != '9999' AND lar_2019.loan_amount != '99999'

);


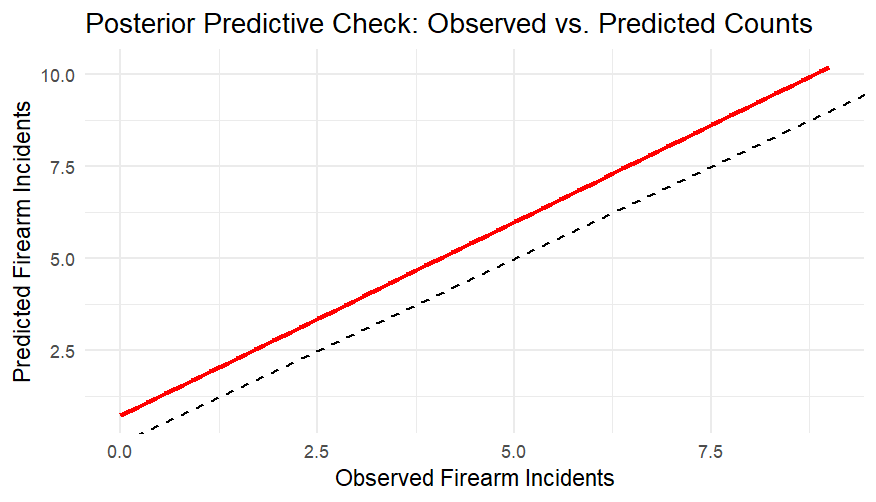


**Supplementary Figure 1**: Posterior Predictive Check for Firearm-Related Homicides. The plot compares observed and predicted firearm-related homicide counts at the census tract level. The black dashed line represents the ideal 1:1 fit, indicating perfect agreement between predictions and observations, while the red line shows the actual trend from the model. The strong alignment between observed and predicted values suggests that the Poisson BYM model provides reasonable estimates of firearm-related violence risk across census tracts.
